# Elevated frequencies of CD8 T cells expressing PD-1, CTLA-4 and Tim-3 within tumour from perineural squamous cell carcinoma patients

**DOI:** 10.1371/journal.pone.0175755

**Published:** 2017-04-19

**Authors:** Richard Linedale, Campbell Schmidt, Brigid T. King, Annabelle G. Ganko, Fiona Simpson, Benedict J. Panizza, Graham R. Leggatt

**Affiliations:** 1 The University of Queensland Diamantina Institute, The University of Queensland, Translational Research Institute, Brisbane, Australia; 2 Department of Otolaryngology—Head and Neck Surgery, Princess Alexandra Hospital, Brisbane, Australia; 3 The University of Queensland Faculty of Medicine, Brisbane, Australia; King's College London, UNITED KINGDOM

## Abstract

Perineural spread of tumour cells along cranial nerves is a severe complication of primary cutaneous squamous cell carcinomas of the head and neck region. While surgical excision of the tumour is the treatment of choice, removal of all the tumour is often complicated by the neural location and recurrence is frequent. Non-invasive immune treatments such as checkpoint inhibitor blockade may be useful in this set of tumours although little is understood about the immune response to perineural spread of squamous cell carcinomas. Immunohistochemistry studies suggest that perineural tumour contains a lymphocyte infiltrate but it is difficult to quantitate the different proportions of immune cell subsets and expression of checkpoint molecules such as PD-1, Tim-3 and CTLA-4. Using flow cytometry of excised perineural tumour tissue, we show that a T cell infiltrate is prominent in addition to less frequent B cell, NK cell and NKT cell infiltrates. CD8 T cells are more frequent than other T cells in the tumour tissue. Amongst CD8 T cells, the frequency of Tim-3, CTLA-4 and PD-1 expressing cells was significantly greater in the tumour relative to the blood, a pattern that was repeated for Tim-3, CTLA-4 and PD-1 amongst non-CD8 T cells. Using immunohistochemistry, PD-1 and PD-L1-expression could be detected in close proximity amongst perineural tumour tissue. The data suggest that perineural SCC contains a mixture of immune cells with a predominant T cell infiltrate containing CD8 T cells. Elevated frequencies of tumour-associated Tim-3^+^, CTLA-4^+^ and PD-1^+^ CD8 T cells suggests that a subset of patients may benefit from local antibody blockade of these checkpoint inhibitors.

## Introduction

Squamous cell carcinoma (SCC) and basal cell carcinoma (BCC) of the skin are the most common forms of cancer with head and neck tumours being particularly prevalent [1]. Development of primary SCC is frequently associated with exposure to ultraviolet radiation resulting in DNA damage amongst other alterations to the epithelial cells (keratinocytes) of the skin. While surgical resection is often successful in eliminating the primary tumour, metastasis of the tumour to secondary sites represents a major complication of aggressive disease. One such metastasis, perineural spread of malignancy along the trigeminal (V) and facial nerves (VII), is a complication of head and neck tumours which is becoming more frequently recognised and results in a poor prognosis for the patient [2, 3]. Diagnosis of perineural spread involves a variety of imaging techniques, particularly MRI, but is often delayed due to the slow development of clinical symptoms [4, 5]. Successful imaging of the tumour is important in determining therapeutic options which include surgical resection and/or radiation treatment [6–8]. While many studies have focused on the immune system in head and neck SCC, very little is known about the local role of the immune system in attacking tumour which spreads along large named nerves [9–11]. One study has shown the expression of FoxP3, a molecule associated with regulatory T cells and immune suppression, in cutaneous SCC is a poor prognostic factor for the development of perineural invasion [12]. Histology is routinely performed to aid in confirmation of perineural tumour spread but reporting on immune infiltrates within the perineural tumour mass is less frequent. A recent immunohistochemistry study from our group showed that both T and B cell infiltrates exist within perineural tumours and that expression of galectin-1 might be associated with poor prognosis [13]. The presence of a T cell infiltrate does not guarantee tumour clearance given the many immunosuppressive mechanisms employed by cancer[14]. Recent interest has focused on inhibitory surface receptors present on T cells, which, when engaged by cells within the tumour microenvironment, leads to in reduced function of the T cell and tumour escape [15]. Successful human trials of PD-1/CTLA-4 blocking antibodies have demonstrated the great potential of these agents in tumour immunotherapy [16, 17]. This success has also promoted the search for other immunomodulatory receptors on T cells including the identification of Tim-3 which negatively regulates T cell function [18, 19].

In the current study, we have assessed the immune cell infiltrate in freshly, excised perineural tumours using flow cytometry. We find that CD8 T cell infiltrates are prominent in the tumour tissue and that patients can express an elevated fraction of PD-1, CTLA-4 or Tim-3-expressing T cells. In addition, both PD-1 and PD-L1 can be co-located within the tumour tissue as demonstrated by immunohistochemistry. This suggests that negative regulators of immunity may contribute to the tumour growth in a subset of patients with perineural spread of SCC.

## Materials and methods

### Patients

Tumour tissue and a blood sample (10mL) were collected at the time of surgery with the main specimen being retained for routine histological confirmation of the tumour by the Pathology department and the sampled tissue utilised for flow cytometric analysis. Over the period of the study, 17 patient samples were collected but one patient was excluded on the basis of a histological diagnosis of neuronitis. Patient written consent was obtained for the research use of tumour samples/blood and this consent procedure and collection of samples was approved by the Princess Alexandra Hospital and University of Queensland Human ethics committee (Approval No. HREC/03/QPAH/197 SSA/13/QPAH/47 and HREC/99/QPAH/34).

### Tumour tissue and blood processing

Blood was collected into heparin-containing tubes to prevent clotting. These cells were then diluted into complete RPMI media (RPMI + penicillin/streptomycin/glutamine + 2-mercaptoethanol + 10% heat inactivated fetal calf serum (FCS)) and layered over Histopaque 1077 (Sigma-Aldrich, St. Louis, USA) for a 30 min spin at 190 *x g*. The interface containing lymphocytes was carefully collected and washed several times in PBS/ 2% heat inactivated FCS before counting cells using a haemocytometer. Freshly collected tumour tissue was placed in complete RPMI media and crushed through a 70 μm strainer to obtain a single cell suspension. The cell suspension was washed in media before resuspending in PBS/2% FCS and counting cells using a haemocytometer. Up to 1 million cells from either the blood or tumour were stained for 1 hr at 4°C with the following fluorescently-labelled antibodies (Biolegend, San Diego, USA) split into two panels: Panel A—anti-human CD3 FITC (clone HIT3a), anti-human CD45 PECy7 (clone HI30), anti-human CD8 PE (clone HIT8a), anti-human Tim-3 BV421 (clone F38-2E2), anti-human CTLA-4 APC (clone L3D10) and anti-human PD-1 APCCy7 (clone EH12.2H7) or Panel B–anti-human CD45 PECy7, anti-human CD3 FITC, anti-human CD19 APC (clone HIB19), anti-human CD56 BV421 (clone HCD56), anti-human TCRVα24 PE (clone 6B11). Matched fluorescently-labelled isotype controls for the Tim-3, PD-1 and CTLA-4 antibodies were also substituted within the panels. Following staining, the cells were washed with PBS/FCS at least two times before being fixed in 5–10% formalin.

### Flow cytometry

Fluorescence data from fixed cells was acquired on a BD LSRII flow cytometer using BDFACSDiva software. Acquired data was analysed using FlowJo software.

### Immunohistochemistry

Immunohistochemistry was performed on the Ventana Discovery Ultra auto-staining platform (Ventana Medical Systems, Inc. USA). Protocol optimisation was performed using appropriate positive control human tissue for each antibody. The antigen retrieval method used was heat retrieval with CC1 retrieval solution (pH 8.0) for anti-PD-1 and ant-PD-L1. Primary antibody concentrations for IHC were PD-1 (NAT105) pre-diluted and PD-L1 (SP263) pre-diluted and incubation was 1 hour at 36°C. The Ventana anti-HQ HRP detection system was used with pre-dilute IgG anti-mouse or anti-rabbit HRP-conjugated secondary antibodies incubated for 1 hour at room temperature. Colour development with DAB reagent and haematoxylin counter-stain were also automated on the platform. Slides were mounted and cover-slips applied by the TRI Histology Core Facility. The negative control included omission of the primary antibody (secondary only control). Imaged were scanned using the Virtual Slide Microscope (VS120-S6-W).

### Statistics

The dataset was analysed using Graphpad Prism. Statistical differences were determined using non-parametric tests with a probability <0.05 being considered significant.

## Results

### Patient characteristics and evaluation of T cell numbers

In this study we have analysed a cohort of 16 patients with perineural spread of cutaneous squamous cell carcinoma (Table 1). The patients were mainly male (14/16) with an average age of 70.2 years. The perineural tumour mass was spread across multiple nerves with 4 patients experiencing recurrent disease.

**Table 1:** Patient characteristics.

Patient characteristics	Observations
Mean age at clinical diagnosis	70.2 years (SD = 9.6; Range = 49.8–83.7)
Sex	14 Male/ 2 Female
Laterality	6 Left/10 Right
Recurrence	4/16 patients
Diagnosis to Recurrence	Mean = 16.3 months (SD = 8.1; Range = 7.5–27.1 months)
Patient Follow up time	Mean = 15.8 months (SD = 8.1; Range = 5.7–34.3 months)
Nerve Involvement	V1 (4); V2 (6); V3 (4); VII (6); Vidian (1); Cervical Plexus (1)
Zone Involvement^#^	Zone 1 (6), Zone 2 (8), Zone 3 (2)
Histology	SCC (15), High Grade Salivary Carcinoma (1)
Margin^	Involved (12)*, Clear/Close (4)

SCC = squamous cell carcinoma.

*All patients with recurrence had involved margins

^#^Zones described in [8]

^ Involved margins indicate residual tumour cells observed on histopathological section after surgery

**Fig 1:**
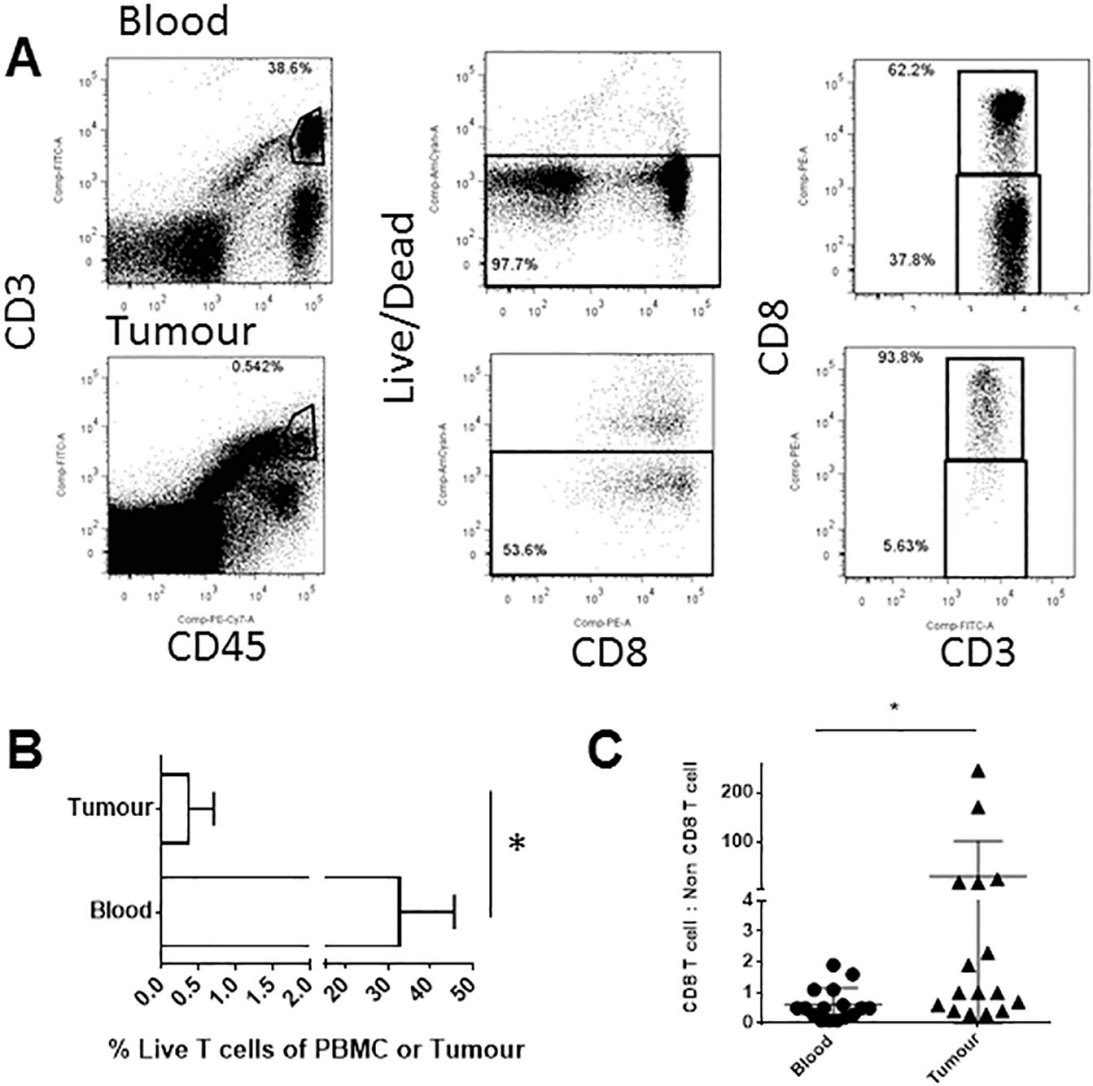
Perineural tumour contains both CD8 and non-CD8 T cells. Freshly excised tumour tissue and blood from each patient was processed into a single cell suspension, stained with antibodies and analysed via flow cytometry. (A) An example of the gating strategy used to distinguish CD8 and non-CD8 T cells within tumour and a blood sample from the same patient. (B) The percentage of live, CD45^+^CD3^+^ T cells within the tumour or blood cells from the cohort of patients is expressed as the mean and SEM. (C) Amongst CD45^+^CD3^+^ T cells in the blood and tumour tissue, the ratio of CD8^+^ to non-CD8 cells was calculated for individual patients. * indicates p<0.05

Our previous study suggested that T cells were present within perineural tumour sections as demonstrated by immunohistochemistry for the T cell marker, CD3 [13]. To quantitatively dissect T cells (and other immune cells) present in a cohort of perineural SCC patients, we have used flow cytometric analysis of gated live cells obtained from paired, fresh blood and tumour tissue samples (Fig 1A). Using 16 patients, we now estimate that the mean T cell frequency among live cells derived from the tumour sample is 0.4% compared with 32.5% T cells within the live PBMCs (Fig 1B). Amongst the T cell fraction, the ratio of CD8 T cells to non-CD8 T cells was low in the blood of the majority of patients (Fig 1C). This is consistent with a general predominance of CD4^+^ T cells over CD8^+^ T cells in the blood of humans under physiological conditions. In contrast, at least 5/16 patients had high CD8:non-CD8 T cell ratios within cells isolated from tumour tissue. This did not appear to correlate with any measured clinical parameters although the sample size is small (data not shown).

### Frequency of different lymphoid lineage cells amongst CD45^+^ cells in the tumour

**Fig 2:**
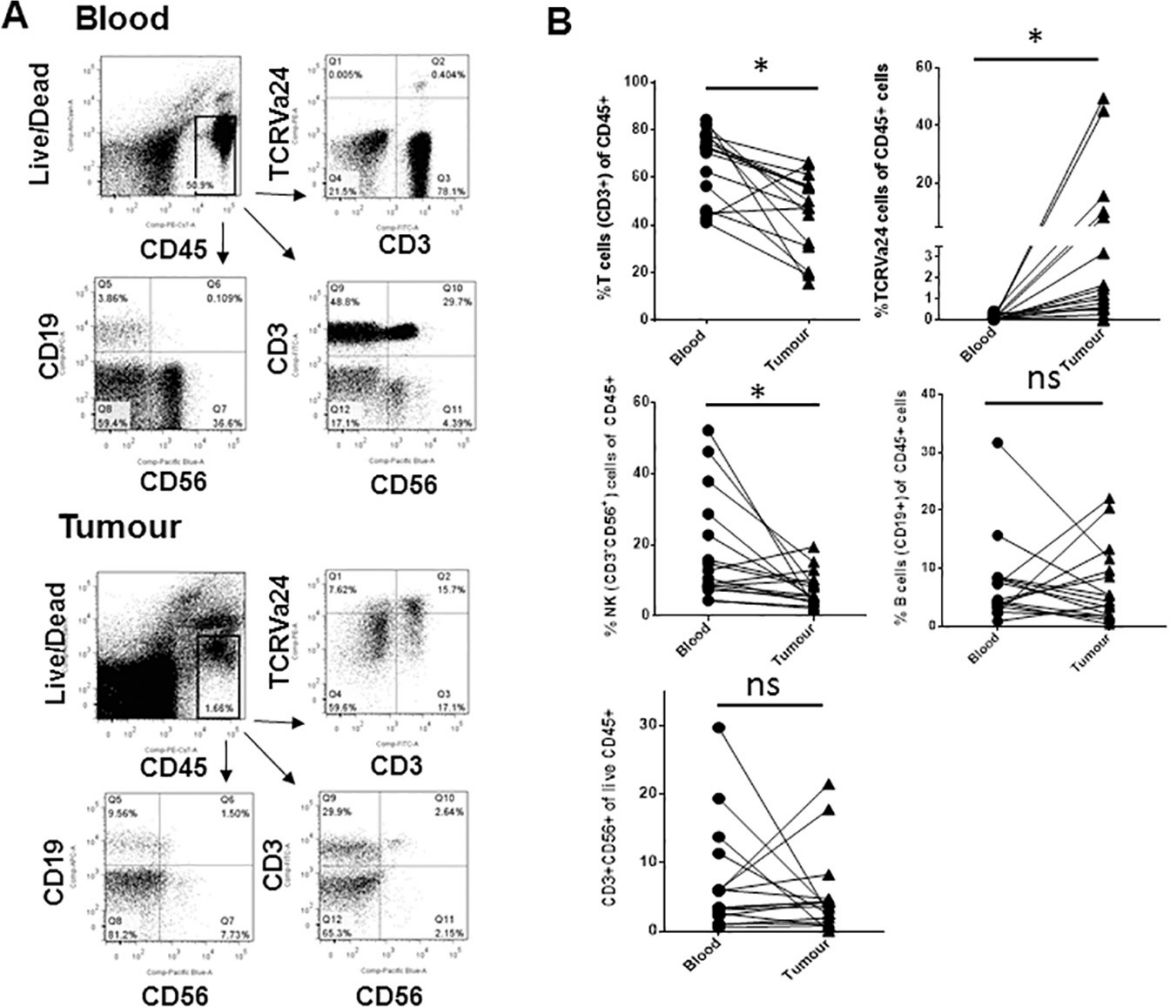
The fraction of T cells, NKT cells and NK cells is significantly different between blood and tumour from patients with perineural SCC. Freshly excised tumour and matched blood samples were stained with antibodies against various immune cell markers and analysed by flow cytometry. (A) An example of the gating strategy used to analyse different immune cell subsets within blood and tumour. (B) The percentage of CD3^+^ (T cells), TCRVa24^+^ (NKT cells), CD3^-^CD56^+^ (NK cells), CD19^+^ (B cells) and CD3^+^CD56^+^ (activated T cells/NKT cells) cells amongst live CD45^+^ cells was plotted for both tumour and blood samples for individual patients. Lines connect the matched blood and tumour sample from each individual patient. * indicates p<0.05

To determine the relative fraction of different lymphoid lineage cells in the blood and tumour tissue, we compared the proportion of CD3^+^ (T cells), CD19^+^ (B cells), CD56^+^ (NK cells) and CD3^+^CD56^+^ cells amongst the CD45^+^ fraction of cells from the blood and tumour (Fig 2A). In both PBMCs and tumour tissue, CD3^+^ T cells were found to predominate amongst the live CD45^+^ cells with most patients having a decreased proportion of these cells in the tumour relative to the blood (Fig 2B). The remaining CD45^+^ cells were found to contain B, NK, NKT and CD3^+^CD56^+^ cells. While many patients had similar proportions of these cell types between the blood and tumour, there were instances of both enrichment and depletion of these cell populations within the tumour. Both B cells and CD3^+^CD56^+^ cells had no significant enrichment within tumour tissue relative to blood while T cells and NK cells were significantly underrepresented within the tumour (Fig 2B). The CD3^+^CD56^+^ cells may contain a mixture of activated T cells and NKT cells. Consequently, to identify conventional NKT cells we employed an antibody against the TCRVα24 chain which is conserved within the conventional NKT cell subset of humans [20]. In contrast to the other cell types, TCRVα24 NKT cells were enriched in tumour tissue relative to blood for the majority of patients (Fig 2B). Together, this suggests that the immune populations being examined in the tumour tissue did not simply reflect the blood composition and that NKT cells are enriched in tumour tissue albeit representing only a small fraction of the immune infiltrate for many patients. Conventional T cells dominate the CD45^+^ tumour infiltrate.

### Expression of inhibitory receptors on T cells

**Fig 3:**
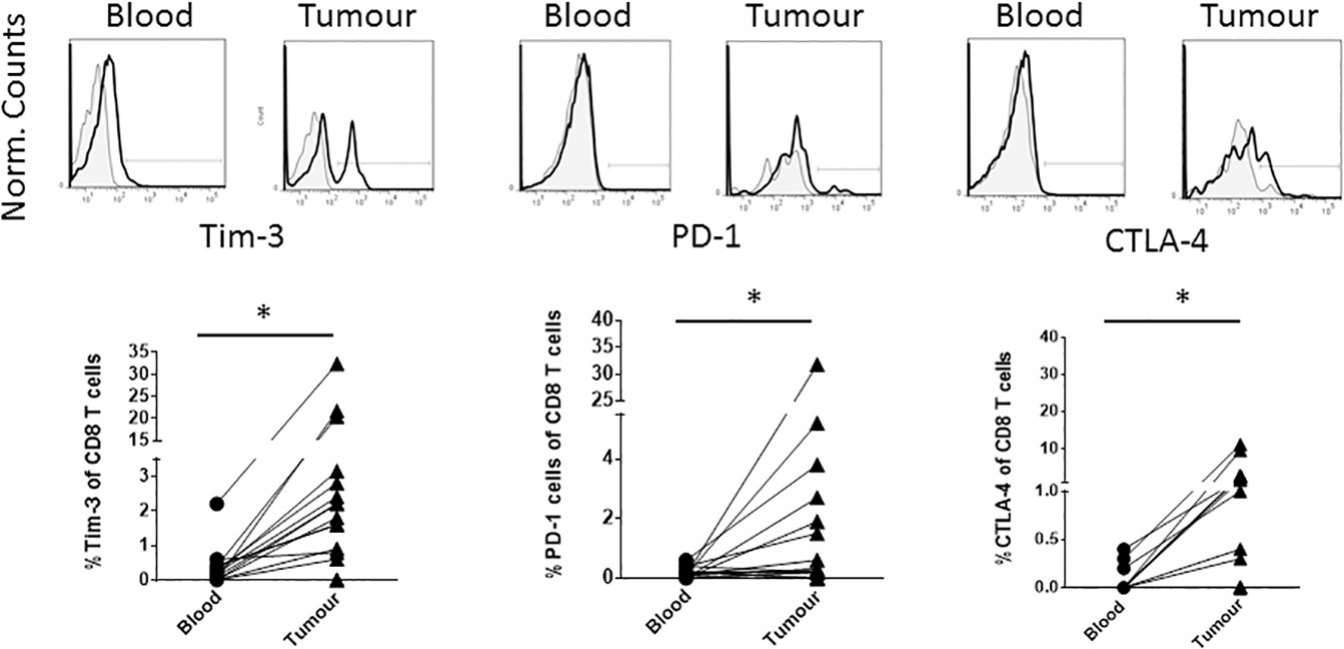
The proportion of CD8 T cells expressing PD-1, Tim-3 and CTLA-4 is elevated in tumour relative to the blood. Upper plots are an example of antibody staining for each indicated receptor after gating on live, CD45^+^, CD3^+^, CD8^+^ cells. Shaded plots represent negative control staining. The lower graphs summarise the percentage of PD-1, Tim-3 and CTLA-4^+^ cells (after subtraction of negative control percentage staining) for individual patients. The lines connect the matched blood and tumour sample from each individual patient. * indicates p<0.05.

**Fig 4:**
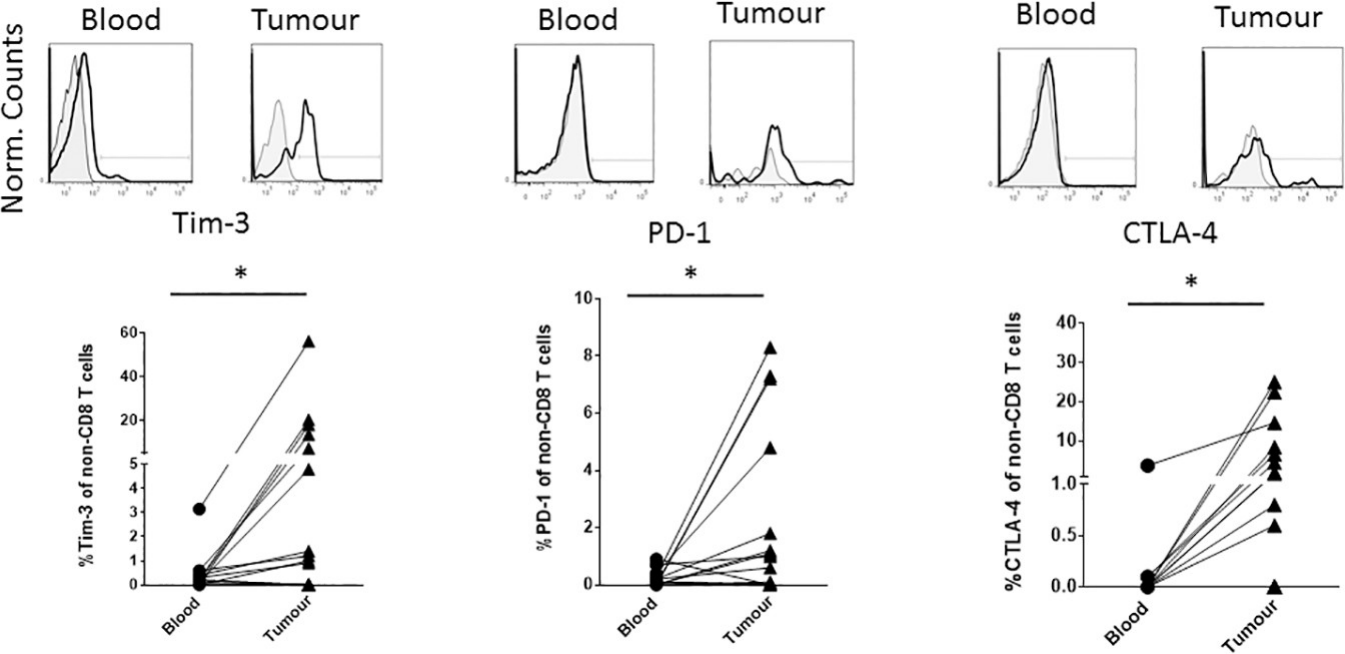
The proportion of non-CD8 T cells expressing Tim-3 and CTLA-4, but not PD-1, is elevated in tumour relative to the blood. Upper plots are an example of antibody staining for each indicated receptor after gating on live, CD45^+^, CD3^+^, CD8^+^ cells. Shaded plots represent negative control staining. The lower graphs summarise the percentage of PD-1, Tim-3 and CTLA-4^+^ cells (after subtraction of negative control percentage staining) for individual patients. The lines connect the matched blood and tumour sample from each individual patient. * indicates p<0.05.

Given that perineural tumours are infiltrated by immune T cells that fail to clear the tumour, we have analysed whether inhibitory receptors such as PD-1, CTLA-4 and Tim-3 are expressed on the surface of tumour-derived T cells using blood as a comparator. Amongst the CD8 infiltrate, the frequency of PD-1, CTLA-4 and Tim-3 expressing cells was significantly elevated in tumour relative to the blood across the patient cohort (Fig 3). One patient had over 30% of the CD8 T cells expressing PD-1 in the tumour relative to a low frequency of PD-1 expression in blood CD8 T cells (1.3%) (Fig 3). Three patients had highly elevated frequencies (greater than 15%) of Tim-3 expressing, intratumoural CD8 T cells (Fig 3). The patient with the highest frequency (32.4%) of Tim-3 expressing CD8 T cells did not co-express either CTLA-4 or PD-1 within the CD8 T cell population (data not shown). Although significantly elevated, the frequency of CTLA-4 expressing cells amongst the CD8 T cell population was generally low (less than 12%) for all patients. Amongst the non-CD8 T cells, significantly elevated frequencies of Tim-3^+^, PD-1^+^ and CTLA-4^+^ cells were again observed in tumour tissue (Fig 4). The patient with the highest intratumoural Tim-3^+^ cell frequency (56.2%) amongst non-CD8 T cells also had the highest frequency of Tim-3^+^ cells (32.4%) amongst CD8 T cells. For both Tim-3 and CTLA-4, there was a correlation between the frequency of CD8 and non-CD8 T cell expression of these markers in tumour tissue (data not shown). No such correlation existed for PD-1.

**Fig 5:**
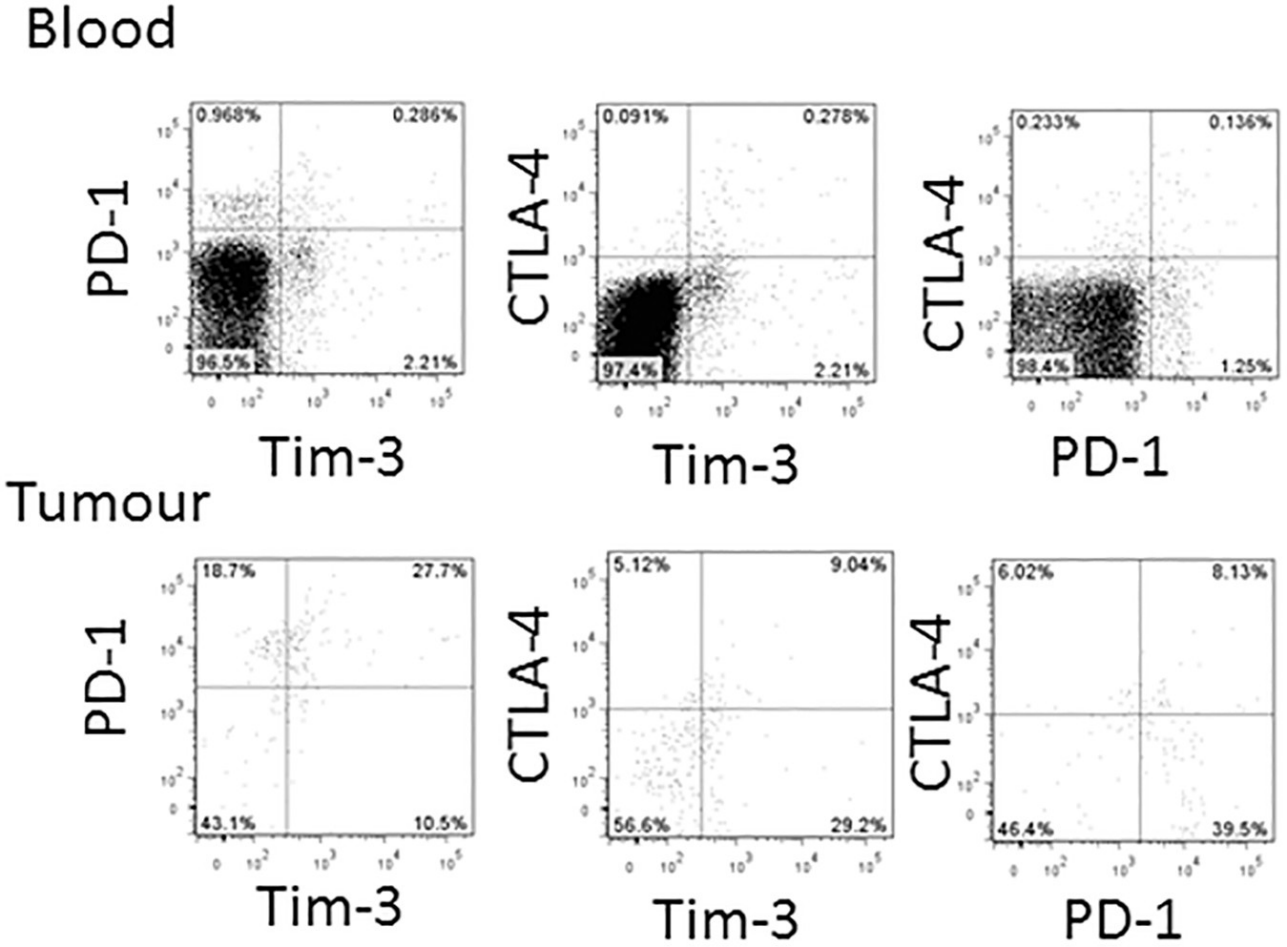
Limited co-expression of PD-1, Tim-3 and CTLA-4 in the blood and tumour of a patient with expression of all three receptors. Gated CD8 T cell populations (live, CD45^+^, CD3^+^, CD8^+^ cells) from the blood (upper plots) and matching tumour sample (lower plots) were analysed for co-expression of PD-1, Tim-3 and CTLA-4 using flow cytometry.

One patient had elevated frequencies of all three inhibitory receptors on CD8 T cells in the tumour but this represented only limited co-expression of PD-1, CTLA-4 and Tim-3 (Fig 5). This suggests that individual CD8 T cells in this patient can express PD-1, Tim-3 and CTLA-4 independently.

**Fig 6:**
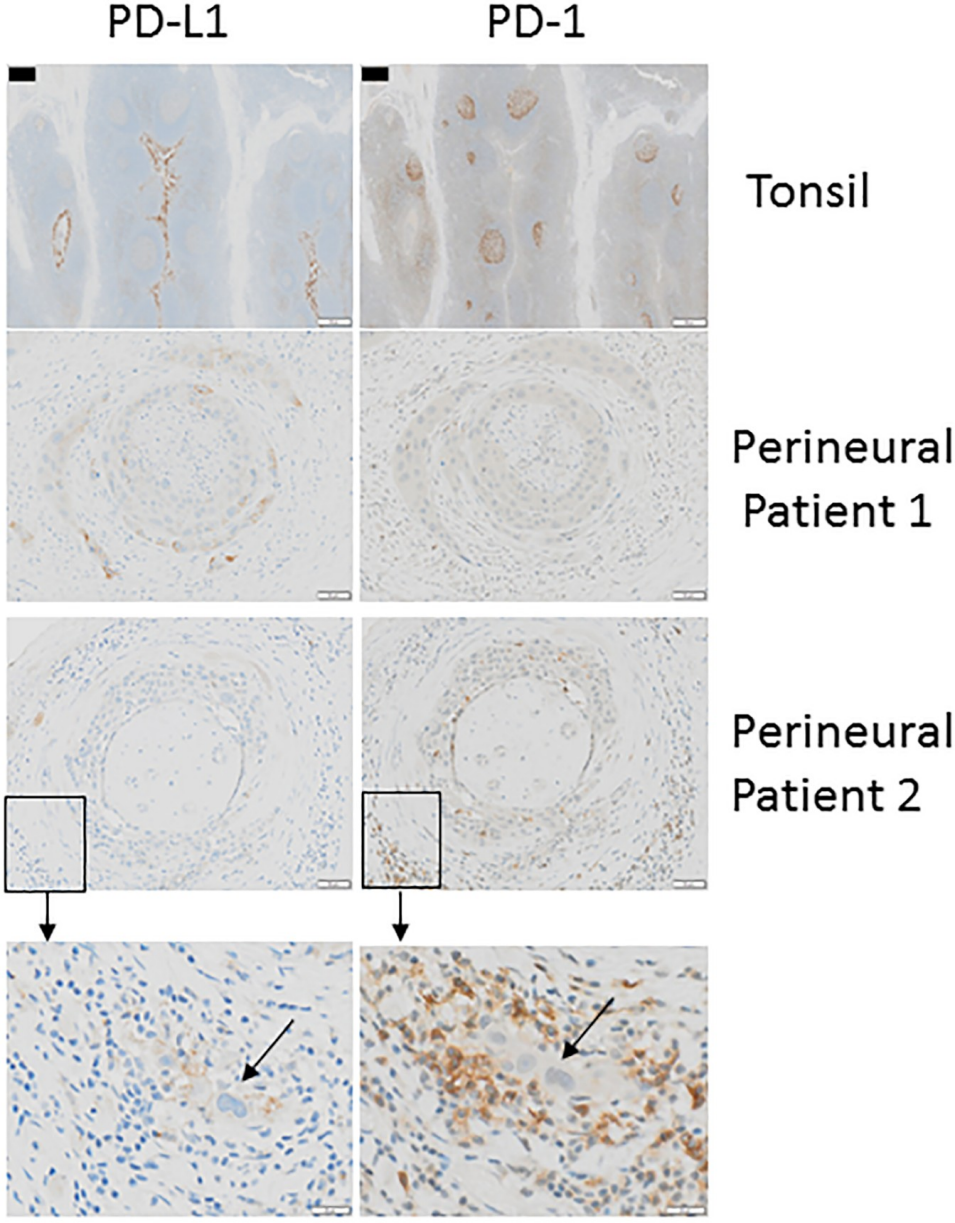
PD-1 and PD-L1 can share a close proximity within the perineural tumour tissue. Tonsil tissue from a healthy patient (upper panel) and perineural tissue samples from two perineural SCC patients (middle and lower panels) were stained with either PD-L1 (left images) or PD-1 (right images) antibodies using immunohistochemistry. Brown colour represents positive staining. Patient 1 was considered to have limited PD-1 staining cells with isolated areas of PD-L1 expression. Patient 2 had high PD-1 staining in isolated patches and light/moderate staining for PD-L1. Boxed areas have been enlarged in the lower figures. Arrows in the bottom panels indicate tumour cells. Scale bars– 100um (tonsil), 50um (Patient 1 and Patient 2), 20um (Patient 2 enlarged).

Finally, we examined the expression of PD-1 and PD-L1 on fixed sections of selected patient tumour tissue by immunohistochemistry to provide spatial information for this inhibitory ligand pair (Fig 6). Tonsil tissue from a patient without perineural disease was used as a positive control and demonstrated strong staining for both PD-1 and PD-L1 (Fig 6-top panels). In selected patients with perineural disease, the frequency of T cells expressing PD-1 in flow cytometry did not necessarily correlate with the immunohistochemical analysis of tumour PD-1 in the same patient. This was perhaps not surprising since the tumour field is unlikely to be homogenous and separate pieces of the tumour were analysed for flow cytometry versus immunohistochemistry. Nevertheless, 3/4 patient samples had PD-1 staining with local foci of PD-L1^+^ tumour cells surrounded by PD-1^+^ inflammatory cells in some patients (Fig 6). This data supports the presence of PD-1^+^ cells in selected patients with perineural disease and suggests that PD-1/PD-L1 can be in close proximity on inflammatory cells and tumour respectively.

## Discussion

Very little is known about the immune response to perineural SCC, although the presence of tumour infiltrating T cells can be a positive prognostic factor in cancer [21]. Our data suggest a dominant infiltrate of both CD4 and CD8 T cells within the perineural tumour mass and yet the tumour continues to grow. This suggests that the intratumoural T cells are unable to engage with the tumour or are being actively immunosuppressed [14, 22]. Immunosuppressive mechanisms within tumours are widely varied but it is interesting that we observed an enrichment of NKT cells which can have a suppressive roles within tumour environments[23]. Our previous mouse study has shown that invariant NKT cells that infiltrated precancerous skin were able to suppress graft rejection via an IFN-g dependent mechanism [24]. In addition, type II NKT cells are known to suppress anti-tumour immunity in mouse models of cancer [25, 26]. Determining the functional role of intratumoural NKT cells within perineural SCC samples will most likely require in vitro co-culture experiments with effector T cells and NKT cells derived from excised perineural tumour tissue. Certainly, there was no apparent relationship between tumour recurrence/progression and the proportion of NKT cells within the tumour mass although larger numbers of patients would be required to increase statistical power.

Another mechanism which reduces the function of intratumoural T cells is checkpoint inhibitory molecules [15]. Best characterised for PD-1 and CTLA-4, these surface receptors on T cells are capable of delivering an inhibitory signal which reduces the anti-tumour function of these cells. Expression of PD-1 on peripheral blood T cells was associated with malignant tumours in ovarian cancer, while high numbers of tumour infiltrating PD-1^+^ immune cells in breast cancer was associated with shorter patient survival [27, 28]. Tumour expression of one of the ligands for PD-1, PD-L1, is also correlated with poor patient outcomes in some cancers including cutaneous squamous cell carcinoma [29, 30]. Antibody blockade of PD-1 and CTLA-4 restores anti-tumour immunity and leads to durable clinical responses in a subset of patients [16]. Our current study suggests an enrichment of PD-1^+^ CD8 T cells in perineural tumour tissue relative to blood although the proportion of intratumoural cells expressing PD-1 was generally low (with the exception of a single patient). This was supported by immunohistochemistry data which showed pockets of PD-1 staining within the tumour sections. Importantly, in one patient, there was clear evidence of PD-L1 expressing tumour cells surrounded by PD-1 expressing inflammatory cells suggesting that a potential interaction between this ligand pair was possible in perineural disease. Further immunohistochemical studies with larger patient numbers would be required to determine the frequency of this colocalisation. Without longitudinal data or greater numbers of patients with recurrent disease, it is also difficult to estimate the proportion of PD-1^+^ cells in flow cytometry or PD-1/PD-L1 expression/co-localisation in immunohistochemistry that would impact the course of disease. Amongst all of the tumour infiltrating T cells, tumour-specific T cells are likely to be infrequent and may correspond with the cells that express PD-1 or other inhibitory molecules. However, tumour antigens are poorly characterised in perineural SCC and therefore distinguishing tumour-specific T cells, using technologies such as fluorescently-labelled peptide/MHC tetramers, is currently difficult for perineural SCC. Certainly, the single patient with >30% PD-1^+^ CD8 T cells might be a good candidate for trialling anti-PD-1 antibody therapy. The expression of CTLA-4 was enriched in both CD8 and non-CD8 T cells in the tumour relative to the blood with varying proportions of CTLA-4^+^ T cells which might predict a widely varied response to checkpoint blockade therapies. It is important to note that both PD-1 and CTLA-4 are being measured at a single timepoint in this study and that expression on cells may vary over time. Our analysis is also confined to surface expression and does not account for any intracellular stores of either receptor.

A third inhibitory receptor on T cells, Tim-3, was also analysed given its emerging role as an inhibitor of T cell function and anti-tumour immunity[31]. Elevated levels of Tim-3 on T cells from osteosarcoma patients have been associated with poor survival while Tim-3^+^PD-1^+^ tumour-specific T cells in melanoma were found to be dysfunctional [18, 32]. Blocking antibodies against Tim-3 have been shown to promote anti-tumour immunity both as sole agents and in combination with other treatments and antibodies against additional inhibitory receptors [33, 34]. Our data suggest that Tim-3 expressing T cells are enriched in perineural tumour and that a subset of patients have a substantially elevated proportions (>15%) of these cells and might be good candidates for targeted antibody therapy. Interestingly, while co-expression of inhibitory receptors on T cells has been reported in the literature, a significant proportion of the analysed cells had single expression of either Tim-3, PD-1 or CTLA-4 [18, 35, 36]. This suggests that expression of each receptor can be independently regulated within the perineural tumour environment.

In conclusion, we have shown that T cells frequently accumulate in perineural SCC lesions where a subset of these cells express PD-1/CTLA-4/Tim-3 not seen in the equivalent blood-derived T cells. This raises the possibility that a subset of tumour infiltrating T cells become dysfunctional within the perineural tumour environment. Future studies should examine the biological function of these T cells and determine whether expression of the inhibitory receptors is altered over time within each patient. Our current data also suggests that a subset of perineural SCC patients might benefit from treatment with blocking antibodies against these checkpoint molecules.
